# Traumatic Experiences and Mental Health of North Korean Refugees in South Korea

**DOI:** 10.4306/pi.2008.5.4.213

**Published:** 2008-12-31

**Authors:** Woo-Teak Jeon, Shi-Eun Yu, Young-A Cho, Jin-Sup Eom

**Affiliations:** 1 Department of Psychiatry, Department of Medical Education, Yonsei University College of Medicine, Seoul, Korea.; 2 Korean Unification Studies, Yonsei University, Seoul, Korea.; 3 Department of Psychology Counseling, Seoul Cyber University, Seoul, Korea.; 4 Department of Psychology, Chungbuk National University, Cheongju, Korea.

**Keywords:** North Korean refugee, North Korean defector, Traumatic experience, Mental health, Personality Assessment Inventory

## Abstract

**Objective:**

This study was conducted at Hanawon-a government sponsored educational facility for the settlement of North Korean refugees during their initial phase in South Korea-in 2004 to explore their mental health status and traumatic experiences in North Korea and during their escape period.

**Methods:**

A survey was conducted in November 2004 with 62 North Korean refugees at Hanawon, and the Trauma Checklist was used to measure their traumatic experiences. To measure their psychological-mental health status, the Personality Assessment Inventory was administered.

**Results:**

In comparison with the traumatic experiences of the North Korean refugees found in the study conducted in 2001 at Hanawon using the same methods, the current study showed a relatively lower frequency of traumatic experiences among the participants. The Personality Assessment Inventory results revealed that the study participants scored higher than average South Koreans in all clinical scales. Particularly, their mania (62.51) and schizophrenia (61.75) scores were above 60, a clinically meaningful score. In the gender comparison, the males exhibited meaningfully higher levels of alcohol problem, non-support, and warmth scale scores.

**Conclusion:**

Compared to the 2001 study, the overall traumatic experiences among North Korean refugees participated in this study. But continous support is necessary for their successful adaptation to South Korean Society have declined. The North Korean refugees at Hanawon experienced difficulties maintaining their mental health and the men in particular requested more intensive care and support for this purpose.

## Introduction

The number of North Korean refugees entering South Korea is continuously on the rise.^1^ Along with the increment in their number, the successful adjustment of the refugees to South Korean society has become an important concern for South Korean society and government.^2^ One of the major factors that influence the social adjustment of North Korean refugees is their mental health. According to the preceding studies on immigrants and refugees, their mental health seems to be the determining factor for their successful integration into a new society.^3^-^6^ Therefore, evaluating the mental health of North Korean refugees and supporting the maintenance of their good mental health should be the focal point of their adjustment to South Korean society. For this purpose, there have been several investigations and studies on the mental health of North Korean refugees.^7^-^13^

The results from these preceding studies revealed that the most decisive factors that affected the mental health of migrants or refugees were trauma and events they experienced during their migrating/escaping periods.^4^-^6^ Several studies on the traumatic experiences of North Korean refugees have been conducted in South Korea.^9^-^11^ Among them, the study by Hong et al.^10^ was the result of research carried out by the current research team. Through several mental health studies, it has been found that North Korean refugees faced challenges in terms of their psychological health. Many of these studies focused on Post-traumatic stress disorder (PTSD),^7^-^14^ while others reported on their psychological health problems in terms of their demographic characteristics, particularly gender.^7^-^9^ Kim et al. conducted a Minnesota Multiphasic Personality Inventory (MMPI) among North Korean refugees^8^ and found that the women exhibited meaningfully higher scores in hypochondriasis (Hs) and masculinity-femininity (Mf) than the men, and that the men exhibited meaningfully higher scores in the depression (D), psychopathic deviate (Pd), psychasthenia (Pt) and social inversion (Si) scales than the women. In the same study, the authors concluded that the male refugees are stricter in their self-assessment, so that they evaluated themselves poorly, showed obsessive behavior, and exhibited more depressive tendencies. Also, among the North Korean refugees, those who were in their twenties, who experienced repatriation to North Korea, and who entered South Korea alone showed more psychologically unstable tendencies than the others.^8^ In their study, in which the Center for Epidemiologic Studies-Depression Scale (CES-D) was used, Han and Lee^10^ reported that the male North Korean refugees group showed a three times higher rate of depression than the female group. In the same study, the authors found that the refugees who were accompanied by their parents or children showed the lowest depression rate.

To make the results of these previous studies clearer and achieve more precise conclusions, this study was designed with the following considerations. First, the investigations need to look into the overall mental health status of North Korean refugees rather than simply concentrating on their PTSD symptom. Also, it is necessary to include more empirical aspects of the North Korean refugees' mental health such as alcoholism and their attitude toward social support/nonsupport. Until now, studies on North Korean refugees mainly have used the MMPI or Symptom Check List-90 (SCL-90) which do not cover these areas.

Second, the mental health studies need to be carried out together with trauma studies due to the strong correlation between one's mental health and traumatic experiences.

Third, investigations of the traumatic experiences of North Korean refugees need to be carried out periodically, because the political and social conditions in North Korea are constantly changing.

Therefore, this study was conducted with the following purposes. First, to comparatively analyze the traumatic experiences of North Korean refugees who entered South Korea in 2004 and 2001 during their time in North Korea and during their escape period. Second, in studying the mental health of the North Korean refugees, to utilize a research method that includes more practical items/scales in its evaluation.

## Methods

### Study group

The participants in this study were 62 North Korean refugees aged between 19 and 55 at Hanawon in November 2004. They all agreed to take part in the survey voluntarily after being explained the purpose of the study.

### Methods of the research

This study was conducted in three parts. First, we investigated the demographic characteristics. The details included in the survey were the age, gender, duration of escape from the time of departure from North Korea to their arrival to South Korea, and their demographic information from North Korea such as their residence, education, marital status, employment status, military experience, and communist party membership.

Second, we evaluated the traumatic experiences of the North Korean refugees by applying a trauma checklist for North Korean refugees. This checklist was developed by Kang^15^ and included the possible psychological traumatic experiences which the North Korean refugees went through while living in North Korea and during their escape period. Our research team had previously used this checklist in the 2001 study^10^ on the traumatic experiences of North Korean refugees and, consequently, it was possible to compare the findings of these two studies.

Third, this study focused on the mental health status of the North Korean refugees. For this purpose, the Personality Assessment Inventory (PAI) was used. The PAI is a self-reporting survey questionnaire developed by Morey which tests objective personal and clinical factors.^16^

Kim et al. standardized the Korean version of the PAI.^17^ This inventory included a total of 344 items and 22 scales. For this study, the concise version with 160 questions^15^ was used. The concise PAI consisted of 21 scales, with the stress scale being excluded. The internal consistency of this PAI was 0.76 and the median test-retest reliability value was 0.79.^17^ The reason for selecting the PAI as the study method is that this inventory includes a variety of scales such as anxiety related disability, suicidal notions, alcohol problems, and drug problems that are closely related to the particularities of the mental health of North Korean refugees, but which are not included in the MMPI or SCL-90. To facilitate the participants' comprehension of the survey questions, 5 North Korean refugees who have resided in South Korea for more than 2 years reviewed the comprehensibility of the Korean version of the inventory.

## Results

### Statistics on demographics

Among the total of 62 participants, 34 (54.8%) were male and 28 (45.2%) were female. The average age of the male participants was 34.75 years (8.15) and that of the female participants was 33.67 years (7.50). The average escape period in a third country for the males was 2.38 years (3.34) and that of the females was 4.41 years (2.66). The geographical background of the participants revealed that the majority (79%, 49 respondents) came from Hamgyong province. As regards the educational level of the participants, 62.9% (11 respondents) graduated from high school. Among the participants, 58.1% responded that they were married, and 32.4% (11) of the male participants said they had military experience. In terms of their party membership, 32.4% (11) of the male participants said they used to belong to the party whereas only 3.6% (1) of the female participants were in the party (Table 1).

### Traumatic experiences

In comparison with the 2001 study, the level of traumatic experiences in North Korea was reduced. In 2001, the percentage of respondents who 'witnessed death from starvation' was 87.4%, but in this study the response to the same question was only 56.6%. Also, in the previous study, 87.4% answered that they had 'witnessed a public execution,' but in 2004, only 43.5% answered that they had witnessed a public execution. For the traumatic experience item of 'experienced great pain from illness or was gravely ill due to lack of treatment', 62.2% answered positively in 2001, but in the 2004 study, the response rate was lowered to 16.1%. However, for the item 'was sent to a corrective facility or prison', 17.3% answered positively in 2001, whereas in 2004, the rate increased to 25.8%. Among the other items, the overall traumatic experiences level decreased (Table 2) compared to the 2001 study.

The percentage of respondents who underwent traumatic experiences during their escape period also decreased in comparison with the results of the 2001 study. For the question 'was in hiding for fear of being found by others,' the responses showed a reduction to 25.8% in 2004 from 83.4% in 2001. Also, those who experienced a 'shortage of food and water to a life threatening level' decreased from 38.6% in 2001 to 17.7% in 2004. The percentage of participants who were 'inspected by North Korean guards or secret agents at the border' was reduced from 52.3% to 17.7%, and that of those who were 'robbed of money, food, and water in their possession' decreased from 23.2% to 4.8%. The level of traumatic experiences the North Korean refugees went through in transit seems to have decreased in 2004 compared to 2001 (Table 3).

### Personality assessment

The results of the PAI for this study are illustrated in Table 4. The score for each scale was calculated in terms of the t-score according to the South Korean adult standard.^17^ The average t-score of the scales, excluding positive impression (m=41.76, SD=12.47) and treatment rejection (m=38.38, SD=11.34), was over 50 points. The positive impression scale consists of items which describe details that deny small defects of self. Generally, if the t-score of positive impression is lower than 58, the participant would not try to unrealistically impress others.^17^ The positive impression of the North Korean refugees (m=41.76, SD=12.47) in this study indicated that they were trying to acknowledge their problems and defects as they were. Also, the t-score for treatment rejection in this study was 38.38 (SD=11.34), which indicated that they had a high motivation for treatment.^17^ When the t-score for treatment rejection is lower than 43, it indicates that the individual acknowledges that she/he has a functional problem and is in need of treatment. Therefore, considering the positive impression and treatment rejection t-scores of the North Korean refugees, it is clear that they acknowledge their problems and defects and that they are highly motivated to participate in treatment and achieve some degree of transformation. In the other 11 clinical scales, the t-scores of the North Koreans refugees were worse than those of average South Korean adults.

The analysis of the PAI results by gender showed that the alcohol problem scale (which evaluates the related behaviors and results of alcohol consumption, abuse, and dependence), non support scale (which examines the perceived lack of social support which includes the availability and quality of social interaction), and warmth scale (which tests the level of rejection and distrust in others and the level of acceptance and empathy in social relations) showed meaningful differences. In the males, the average t-score for the alcohol problem scale was 58.50 (SD=14.24), which was significantly higher than that for the females (m=49.36, SD=8.51). In the case of the nonsupport scale, the average t-score for the male participants was 56.77 (SD=10.41), which was meaningfully higher than that of the female participants (m=50.36, SD=10.02). This result indicated that the perceived social support was lower among the males. The average t-score for warmth among the male participants was 57.00 (SD=11.31) whereas that for the females was 50.84 (SD=10.23), which indicated that the men were "were warmer and more interest in interpersonal relationship than the females (Table 4).

The two clinical scales of mania (m=62.51) and schizophrenia (m=61.75) showed scores above 60 which are above the 85^th^ percentile of the scores for average South Korean adults. To understand the reasons for these results, better the scores for each variable were analyzed (Table 5). The high mania score was due to the high irritability level. This showed that the manic tendency among the North Korean refugees did not come from an increased activity level or excitement, but from irritability or anxiety which might be caused by their new, unfamiliar life in South Korea. The high schizophrenia score was the result of their high social detachment score (m=5.24, SD=2.48), not of a high level of previous psychotic experiences or thought disorder. These results demonstrate that the social isolation of the North Korean refugees during their initial period of settlement might have a bad influence on their mental health.

## Discussion

To correctly understand the results obtained from this study, it is necessary to take into consideration the period of time when the North Koran refugees entered South Korea. The study by Hong et al. was conducted with North Korean refugees who entered South Korea in 2000^10^ and the current study was done with North Korean refugees who entered South Korea in 2004. The target population in the report by Yu was North Korean refugees in protective facilities in China in 2006.^11^ Also Yoon et al. studied 112 North Korean refugees who entered South Korea in 2007 along with 175 other North Korean refugees who entered during 2004-2006.^18^ Considering the different periods of time when the refugees entered South Korea, the results of the present study can be discussed as follows.

First, it was found that the traumatic experiences of North Korean refugees during their residence in North Korea are diminishing. For example, for the variable 'witnessed someone else being executed' in the traumatic experiences category, 87.4% of the respondents in Hong's^10^ 2000 study, 43.5% in the current 2004 study, 40% in the 2006 study by Yu,^11^ and 55% in the 2004-2007 study by Yoon answered that they had experienced this trauma. For the item 'witnessed or heard news of death by starvation of a close neighbor', compared to the 81.3% who answered positively in Hong's 2001 study,^10^ 56.5% of the participants in the current study answered positively, and 52.3% reported that they had experienced this item in Yu's study.^11^ These results might reflect the gradual loss of tight control by the North Korean government over the North Korean population. Actually, the North Korean refugees in Hong et al.'s study in 2000 escaped from North Korea while the country was suffering from its worst food crisis and, consequently, the border controls were very tight. In North Korea, food rationing was completely halted in 1994,^19^-^21^ and Kim Jung Il organized the open execution of defectors in the midst of a national crisis.^22^ On the other hand, the participants of this current 2004 study escaped for the purpose of having a better life during a time when the food condition in North Korea had ameliorated. The domestic situation in North Korea progressed without any large change, and the 2006 and 2007 studies reflect the somewhat weakened social control in the country.

Second, the overall trend in the traumatic experiences of North Korean refugees during the escape period does not show a generally diminished pattern, in contrast with their experiences in North Korea. Rather, it shows instability. The results of the current study showed that the traumatic experiences during the escape period declined compared with Hong et al.'s study in 2000.^10^ However, in the later study by Yu^11^ in 2006, it was reported that the level of traumatic experiences level had increased. For example, in 2000, Hong^10^ reported that 83.4% of the refugees said that they '[were] in hiding for fear of being spotted by others.' In the current study in 2004, 25.8% of the refugees responded positively to this question, and in the 2006 study by Yu,^11^ 53.8% of the participants said they had experienced this trauma. Also, for the item 'Someone who was escaping with me died during the escape,' in 2000 Hong's report^11^ 8.1% and in the 2004 current study 1.6% responded positively, but in 2006,^11^ 6.2% reported that someone they were escaping together with had died in the process. It can be supposed that the smaller sample numbers in the 2004 and 2006 studies than that in the 2001 study by Hong may have influenced the result. Nevertheless, the level of traumatic experiences during their escape has not consistently declined. Instead it has fluctuated according to the political conditions in China and North Korea. Another reason for this fluctuation may be the complicated international relations among North Korea, South Korea, and China, and the subsequent denial of internationally recognized refugee status of North Koreans in China.

Furthermore, despite the massive number of North Korean refugees, the Chinese government's tight control and forced repatriation has been reinforced and, therefore, the human rights condition for North Korean refugees in transit has not improved.^23^

Third, the mental health problems that North Korean refugees exhibit during their stay in Hanawon are manifested in the form of "a normal reaction to abnormal stress." In other words, the psychological problems they show in this period do not mean that they are psychiatrically ill, but rather their problems can be understood as a normal reaction to a sudden life changing event resulting from their entering a completely different world. In fact, despite their high level of traumatic experiences, the only clinical scales that showed meaningfully high scores were mania and schizophrenia. More specifically, their manic and schizophrenic tendencies were not caused by increased activity, grandiosity, psychotic experiences, or thought disorder, but by their inevitable irritability or social detachment due to their entering a completely new society. Therefore, it is important not to discuss the mental health of North Korean refugees as if they were psychologically unhealthy. Kim^8^ also found in her MMPI study that paranoia showed the highest t-scores. The average t-score for men was 56.28 (SD=10.64) and that for women was 55.98 (SD=11.04), but these scores do not indicate psychosis.^24^ However, these results show that North Korean refugees are socially and psychologically detached and irritated. Therefore, the educational programs and counseling programs in Hanawon should focus on solving these issues.

Fourth, the study results indicated that there were differences in the mental health status according to gender, so it is important to provide properly customized support for men and women. Among the statistical variables that showed meaningful deviations were alcohol problems in the clinical scale, nonsupport in the treatment scale, and warmth in the interpersonal scale. Clinically, the men were more vulnerable to alcohol problems, and since this could negatively influence their life at work and at home, more aggressive education on alcohol problems is suggested. In the treatment scale, it was found that the men perceived that they received less social support. It is recommended that men need education and explaination about the social support system and resources in South Korean society in more detail which can provide with more moral support.

It was also found in the previous studies by Cho^7^ and Kim and Jeon^8^ that men show a more depressive tendency than women. Therefore, it is important to provide psychological support for men. Warmth scale (one of the interpersonal scales) is a scale that conceptualizes the spectrum of interpersonal relationships from warm to cold, and those who score high on this scale are thought to be warm and have more interest in interpersonal relationship, whereas those who score low are thought to be cold and dismissive. The female members of the North Korean refugees showed a similar level of warmth of South Korean females, whereas the male North Korean refugees showed a higher level of warmth than male South Koreans, and such results demonstrate the differences between the genders. In other words, the men may feel less for social support, but in interpersonal relationships they accept others more readily and aspire to make social connections. Therefore, the development of programs that can facilitate the successful social adjustment of North Korean refugees is called for.

The results of this study demonstrate that North Korean refugees have experienced many severely traumatic events which hinder their successful adaptation into South Korean society. To facilitate the successful adjustment of North Korean refugees to life in South Korea, it is important to provide them with continual support and understanding from the viewpoint of their mental health.

## Figures and Tables

**TABLE 1:**
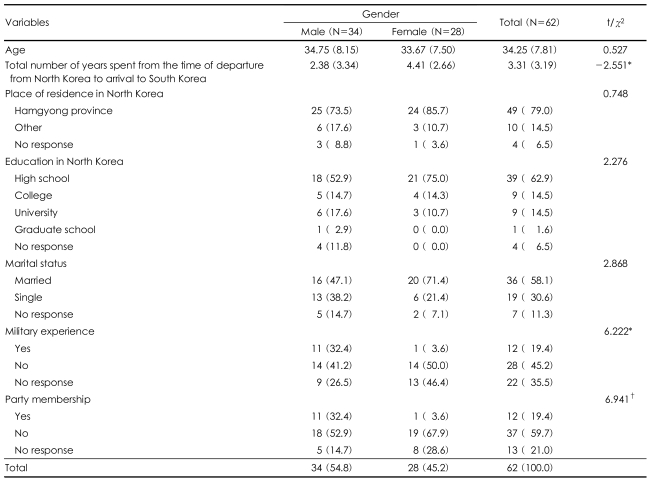
Demographic characteristics of the participants by gender

^*^The parenthesis indicates percentage, ^†^No response was excluded from the χ^2^ calculation

**TABLE 2:**
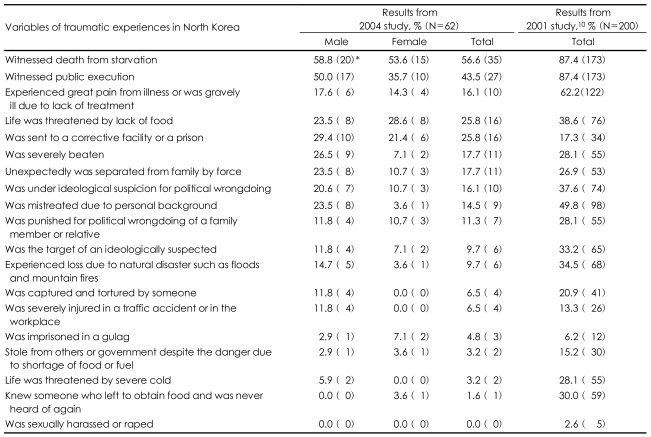
Traumatic experiences in North Korea

^*^The numbers in parenthesis indicate the number of respondents

**TABLE 3:**
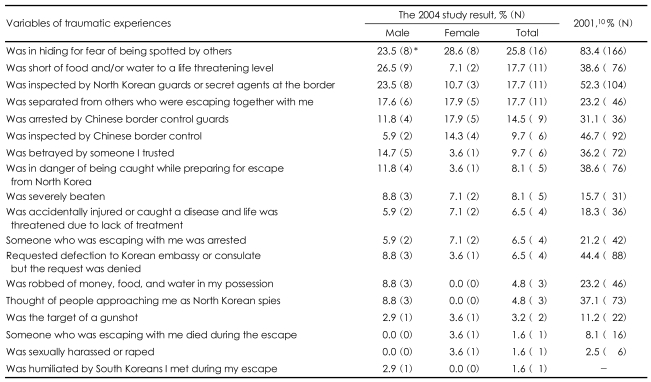
Traumatic experiences during the escape period

^*^The number of parenthesis indicate the number of respondents

**TABLE 4:**
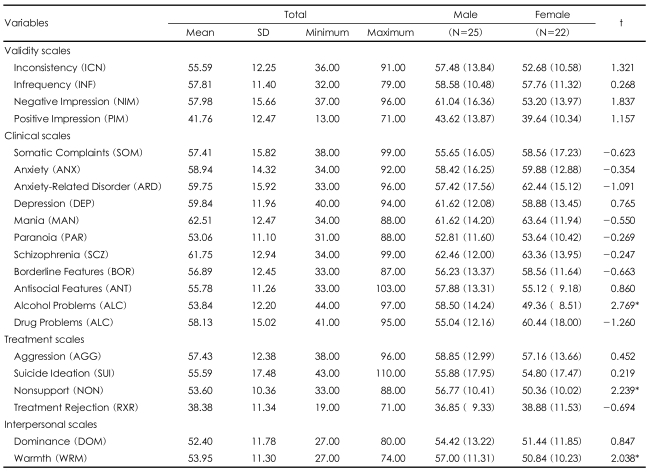
The average means for the PAI scales for the personality assessment

^*^p<0.05. PAI: personality assessment inventory, SD: standard deviation

**TABLE 5:**
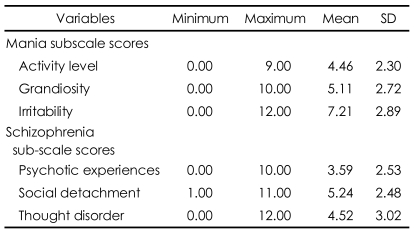
Detailed sub-scale scores of depression and schizophrenia among PAI scales

PAI: personality assessment inventory, SD: standard deviation
